# Case Report: A rare case of fumarate hydratase (FH)-deficient uterine leiomyoma in a 39-year-old woman: a sentinel finding prompting hereditary cancer syndrome evaluation

**DOI:** 10.3389/fonc.2026.1904858

**Published:** 2026-08-05

**Authors:** Nan Li, Li He

**Affiliations:** 1 Department of Gynecology, Ningbo Key Laboratory for Reproductive Health in Major Gynecological Diseases, Women and Children's Hospital of Ningbo University, Ningbo, China; 2 Ningbo Municipal Key Laboratory of Major Gynecological Diseases and Reproductive Health, Ningbo, China; 3 Department of Gynecology, People’s Hospital of Kuqa, Kuqa, China

**Keywords:** case report, fumarate hydratase (FH), hereditary leiomyomatosis and renal cell carcinoma (HLRCC) syndrome, immunohistochemistry, severe anemia, uterine leiomyoma

## Abstract

**Background:**

Fumarate hydratase (FH)-deficient uterine leiomyoma is a rare pathologic marker for hereditary leiomyomatosis and renal cell carcinoma (HLRCC) syndrome. Its recognition is crucial for initiating life-saving surveillance.

**Case presentation:**

We report the case of a 39-year-old woman from a minority ethnic background presenting with a pelvic mass and progressive heavy menstrual bleeding culminating in severe anemia (hemoglobin 5.8 g/dL). Imaging revealed numerous uterine leiomyomas. She underwent an open myomectomy. Histopathological examination confirmed the diagnosis of FH-deficient leiomyoma, supported by immunohistochemistry showing complete loss of FH expression and strong positivity for 2-succinocysteine (2SC). This pathological finding served as a sentinel event, prompting clinical evaluation for hereditary leiomyomatosis and renal cell carcinoma (HLRCC) syndrome, regardless of germline status.

**Conclusion:**

This case highlights that the presentation of multiple leiomyomas with rapid symptom progression in a young woman should raise clinical suspicion for an underlying syndromic association. Pathologic confirmation via FH/2SC immunohistochemistry is pivotal and alone warrants initiation of renal surveillance, as recommended by current guidelines. The diagnosis mandates a multidisciplinary strategy involving genetic counseling, offering germline FH mutation testing, and proactive renal surveillance, thereby shifting management from a surgical problem to comprehensive cancer prevention.

## 1 Introduction

Uterine leiomyomas are exceedingly common, but a distinct minority, characterized by deficiency in the tricarboxylic acid cycle enzyme fumarate hydratase (FH), carries profound clinical implications. FH-deficient leiomyomas are strongly associated with HLRCC, an autosomal dominant cancer predisposition syndrome caused by germline mutations in the *FH* gene, although somatic biallelic inactivation of FH can also give rise to identical tumor pathology. Women with HLRCC often develop early-onset, multiple, and symptomatic leiomyomas, which can be the first manifestation of the syndrome (1, 2). The diagnosis, established by loss of FH expression on immunohistochemistry (IHC) and concurrent positivity for 2SC, serves as a critical sentinel event, triggering evaluation for aggressive papillary type 2 renal cell carcinoma (3, 4). We present a detailed case of a woman from Xinjiang, China with a rapidly symptomatic pelvic mass, leading to the diagnosis of an FH-deficient leiomyoma, to highlight the diagnostic pathway, underscore the associated morbidity, and emphasize the imperative multidisciplinary follow-up in a resource-limited setting.

## 2 Case presentation

### 2.1 Patient information

A 39-year-old Uyghur woman, gravida 2 para 2 (with one previous vaginal delivery and one lower-segment cesarean section), presented with a chief complaint of a pelvic mass discovered 5 months prior and increased menstrual flow for 3 months. She had a copper intrauterine device in place for 7 years. Her menstrual history was notable for menarche at age 12, with previously regular cycles (6–7 days duration, using 7–8 regular pads per day). Her family history was non-contributory for renal cancer, cutaneous leiomyomas, or multiple uterine fibroids. Her parents and one sister were reportedly healthy. Her ethnicity is mentioned solely to contribute to the sparse data on HLRCC-associated conditions in underrepresented populations and does not imply any ethnic-specific predisposition.

### 2.2 Clinical findings

Physical examination revealed a firm, irregular, and enlarged uterus palpable approximately 4–5 cm above the symphysis pubis. No cutaneous lesions were noted.

Laboratory investigations at her emergency presentation revealed severe microcytic hypochromic anemia with a hemoglobin level of 5.8 g/dL. Upon admission for surgery, her hemoglobin was 8.9 g/dL after transfusion.

Transvaginal ultrasound confirmed a markedly enlarged, irregular uterus containing 8–10 well-defined hypoechoic nodules. The largest, located on the anterior wall, measured approximately 63 x 66 mm with peripheral ring-like vascularity on color Doppler. An intrauterine device was noted in a normal position.

### 2.3 Timeline

The diagnostic timeline vividly illustrates the rapid progression of the patient’s condition. The course began five months prior to admission when a pelvic mass was incidentally discovered on a routine ultrasound at a local health center, for which she sought no further evaluation. Three months prior to admission, she experienced the onset of marked heavy menstrual bleeding. Approximately 2.5 months prior, she presented to our outpatient clinic where ultrasound confirmed multiple uterine leiomyomas; hospitalization was recommended but was declined by the patient at that time. Several days later, her symptoms culminated in a visit to the emergency department with severe anemia (hemoglobin 5.8 g/dL), necessitating blood transfusion. Her current presentation was for elective surgical management, with a preoperative hemoglobin level of 8.9 g/dL. A graphical representation of this timeline is provided as **Supplementary Figure 1**.

### 2.4 Diagnostic assessment

Preoperatively, the differential diagnoses included multiple typical uterine leiomyomas, adenomyosis, and, given the rapid symptom progression, the remote possibility of a uterine sarcoma. However, the patient’s relatively young age, the multiplicity of fibroids, and the severity of anemia raised clinical suspicion for a syndromic association. An open abdominal myomectomy was chosen over hysterectomy because the patient strongly desired uterine preservation and retained fertility potential; a minimally invasive approach was deemed unsuitable due to the large number and size of the fibroids, the presence of a prior cesarean scar, and the anticipated intra-abdominal adhesions. The preoperative clinical diagnoses were: 1) Multiple uterine leiomyomas, 2) Uterine scar from previous cesarean section, 3) Severe anemia (post-transfusion), and 4) Intrauterine device *in situ*.

### 2.5 Therapeutic intervention

On January 15, 2026, an open abdominal myomectomy with lysis of intestinal adhesions was performed. Intraoperatively, the uterus was enlarged equivalent to a 16-week gestation, with a bosselated surface featuring multiple subserosal protrusions. The largest leiomyoma (approx. 5 x 6 x 6 cm) was enucleated from the anterior wall. Additional nodules (~3 cm) were removed from the fundus and left posterior wall. Dense adhesions between the right adnexa, sigmoid colon, and omentum were lysed. The left adnexa appeared normal. The surgery lasted 150 minutes with an estimated blood loss of 400 mL.

### 2.6 Pathological findings

**Figure 1:**
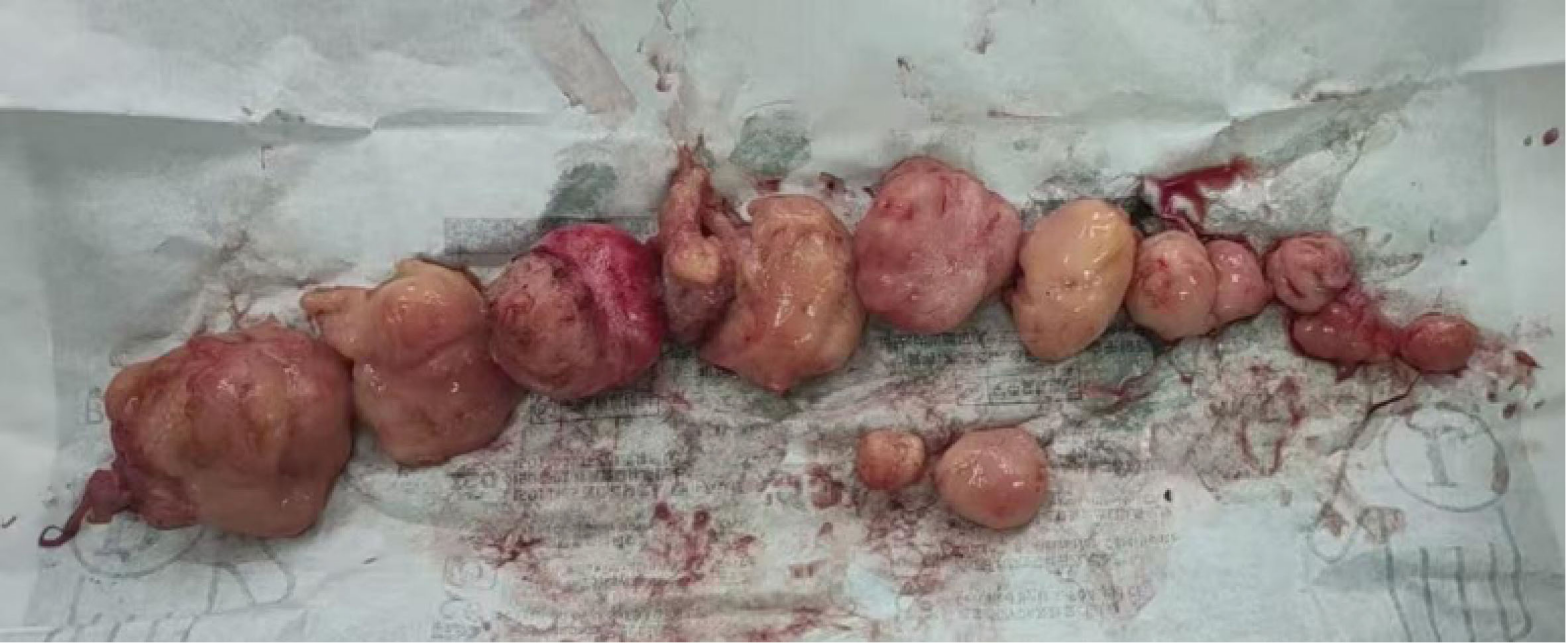
Gross appearance of the resected leiomyomas. The aggregated nodules measured 10 x 9.5 x 4 cm in totality. The cut surface of a representative large nodule demonstrates a characteristic whorled, firm, and white-tan appearance.

Gross examination (**Figure 1**) of the submitted specimens, which collectively formed a conjoined, nodular mass measuring 10 x 9.5 x 4 cm, showed a whorled, firm, white to tan cut surface. The individual nodules were well-circumscribed.

**Figure 2:**
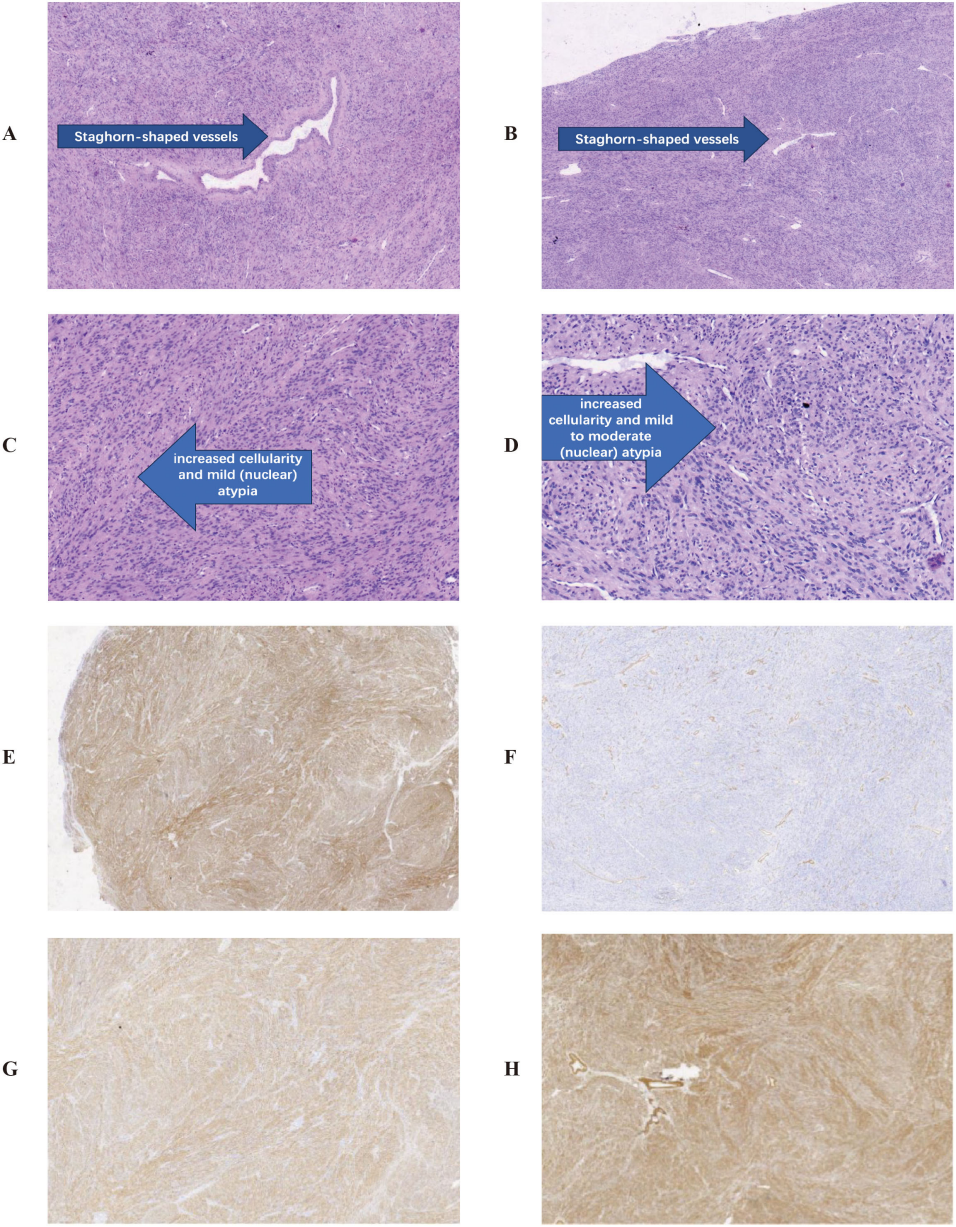
Histopathological and immunohistochemical features of the FH-deficient leiomyoma. **(A)** Hematoxylin and eosin (H&E) staining shows prominent staghorn-shaped (hemangiopericytoma-like) vessels within the tumor stroma. **(B)** Another H&E field highlights scattered staghorn-shaped vessels (arrow). **(C)** H&E staining reveals an area of increased cellularity with mild nuclear atypia. **(D)** H&E staining of a focus with increased cellularity and enlarged nuclei with prominent nucleoli; some cells exhibit subtle perinucleolar clearing (arrows). **(E)** Immunohistochemistry (IHC) for Desmin shows diffuse strong positivity, confirming smooth muscle differentiation. **(F)** IHC for FH shows complete loss of nuclear and cytoplasmic expression in tumor cells. Robust internal positive control staining is present in endothelial cells (arrowhead), validating the assay. **(G)** IHC for 2-succinocysteine (2SC) shows diffuse strong cytoplasmic positivity. **(H)** IHC for smooth muscle actin (SMA) is strongly positive. (Original magnifications: A, B, E-H: 200×; C, D: 400×).

Microscopic examination revealed a smooth muscle tumor with characteristic features suggestive of FH deficiency. Hematoxylin and eosin (H&E) staining (**Figures 2A–D**) showed intersecting fascicles of spindle cells. Key morphological findings included: scattered, prominent staghorn-shaped (hemangiopericytoma-like) vessels (**Figure 2A**); areas of increased cellularity with mild nuclear atypia (**Figure 2B**); foci containing cells with enlarged nuclei and conspicuous nucleoli, some surrounded by a subtle perinucleolar clearing (**Figures 2C, D**). While the classical eosinophilic macronucleoli with distinct perinucleolar halos were not the dominant feature, the constellation of findings was consistent with the spectrum of FH-deficient leiomyoma. No coagulative tumor cell necrosis or significant mitotic activity was observed.

Immunohistochemistry (IHC) was performed (**Figures 2E–H**). The tumor cells showed: complete loss of nuclear and cytoplasmic FH expression, with strongly positive internal control in endothelial cells (arrowhead), confirming the validity of the negative result (**Figure 2F**); diffuse and strong cytoplasmic positivity for 2SC (**Figure 2G**); positivity for SMA (**Figure 2H**) and Desmin (**Figure 2E**), confirming smooth muscle differentiation; a wild-type (scattered) pattern for p53; and a low proliferation index with Ki-67 labeling approximately 1%.

The final pathological diagnosis was FH-deficient leiomyoma.

### 2.7 Follow-up and outcomes

The patient’s postoperative recovery was uneventful, and she was discharged on postoperative day 5.

**Figure 3:**
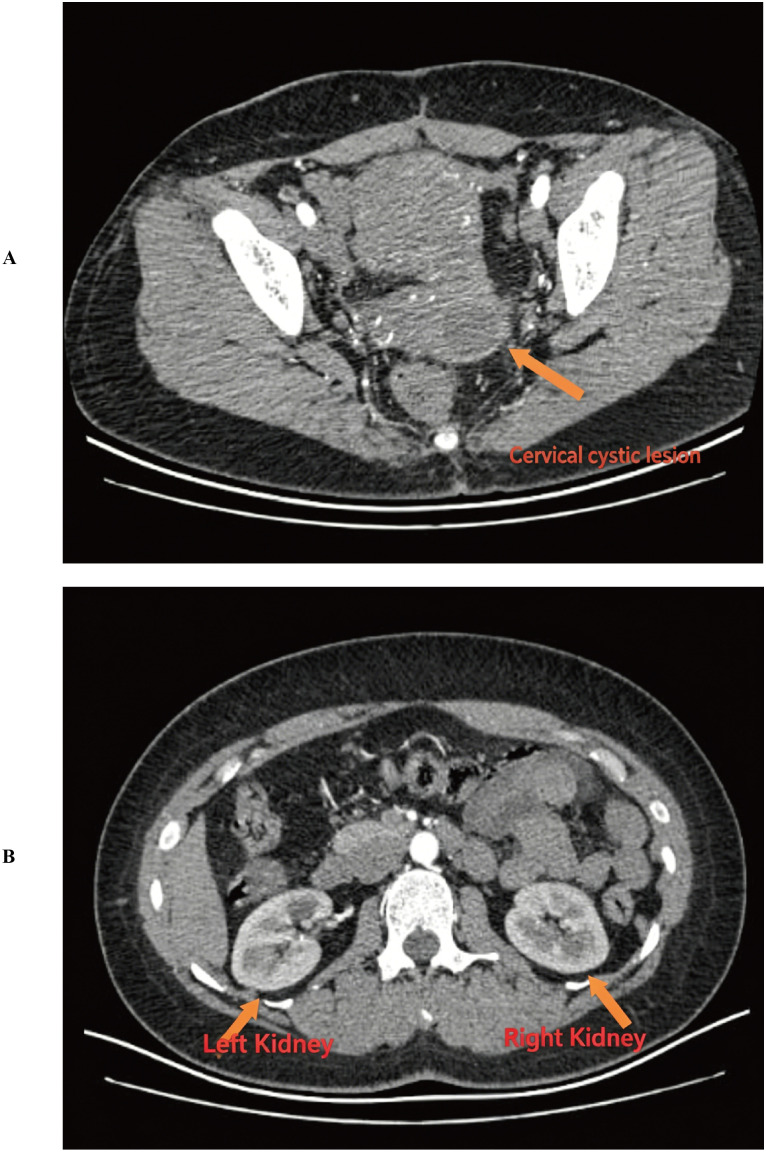
Postoperative contrast-enhanced computed tomography (CT) of the lower abdomen performed 2.5 months after myomectomy. **(A)** Axial image shows a well-defined, non-enhancing cystic lesion (arrowhead) at the left lateral aspect of the cervix, compatible with a benign nabothian cyst. **(B)** Coronal image demonstrates normal morphology of both kidneys (arrows) with no evidence of renal masses or other abnormalities.

A contrast-enhanced computed tomography (CT) scan of the lower abdomen was performed on April 7, 2026 (approximately 2.5 months postoperatively) as a routine follow-up imaging evaluation. The CT scan (**Figure 3**) showed an enlarged uterus with multiple, nodular, heterogeneous enhancing lesions. The enhancement pattern of these nodules was similar to that of the normal myometrium, consistent with residual or recurrent uterine leiomyomas. Given the patient’s asymptomatic status and desire to preserve the uterus, these findings are being managed expectantly with periodic clinical and sonographic assessment. Additionally, a 28-mm non-enhancing cystic lesion was noted at the left lateral aspect of the cervix, compatible with a benign nabothian cyst. No other pelvic abnormalities or evidence of metastatic disease were identified. Formal genetic counseling was provided by a clinical geneticist via telemedicine from Urumqi. The counseling session covered the autosomal dominant inheritance pattern of HLRCC, the 50% risk to first-degree relatives (including the patient’s children and sister), the estimated 15–20% lifetime risk of aggressive renal cell carcinoma, and the recommendation for annual contrast-enhanced renal MRI surveillance beginning at 8–10 years of age for at-risk relatives. The patient demonstrated understanding of the hereditary implications but declined germline FH gene testing due to financial constraints and the long travel distance (the nearest testing facility is approximately 700 km from Kuqa). She expressed concern for her relatives and was willing to inform them, though they have not yet pursued testing. Despite the lack of genetic confirmation, the pathological diagnosis of FH-deficient leiomyoma is, in itself, sufficient to prompt clinical surveillance for HLRCC according to current guidelines (5). She was also advised to undergo a dermatological examination for cutaneous leiomyomas and to inform her first-degree relatives about the potential hereditary risk. A repeat, detailed family history remained negative for related cancers at the time of last follow-up. The patient consented to the recommended annual renal cancer surveillance, which will be followed up at our institution. A long-term follow-up plan has been established, and we intend to report the findings of annual renal MRI and cascade testing in a subsequent publication.

### 2.8 Patient’s perspective

During the counseling process, the patient shared that while the possibility of a hereditary cancer syndrome was initially distressing, she felt reassured by the structured surveillance plan. She cited the prohibitive cost of genetic testing and the 700-kilometer journey to Urumqi as insurmountable barriers at present but expressed a willingness to reconsider testing if circumstances change. She also voiced concern about the potential risk to her children and siblings and stated that she would encourage them to seek medical advice. Her decision to proceed with renal surveillance without genetic confirmation was made after understanding that it is a pragmatic, guideline-supported alternative.

## 3 Discussion

This case vividly illustrates the aggressive clinical potential of FH-deficient leiomyomas and their critical role as a harbinger of HLRCC syndrome. Our patient’s course from an incidental finding to transfusion-requiring anemia within months underscores the significant morbidity these lesions can cause and aligns with reports of rapid growth in some FH-deficient tumors (6). Crucially, while FH deficiency in uterine leiomyomas is most commonly associated with germline mutations in the context of HLRCC, it may also arise from somatic biallelic inactivation of the FH gene without any heritable predisposition. In the present case, without germline testing, the origin of the deficiency remains undetermined; however, the management implications—chiefly renal surveillance—are identical, given the high association with the syndrome (7, 8).

The pathological diagnosis hinges on a high index of suspicion. While classic histologic features like prominent staghorn vessels and eosinophilic macronucleoli with perinucleolar halos are helpful, they are not always present or may be subtle, as in our case (7). In our patient, the presence of staghorn vessels and nuclear atypia in a young woman with multiple tumors prompted the diagnostic IHC workup. The combination of complete FH loss and 2SC gain is now considered the gold standard for diagnosis, offering high sensitivity and specificity (3, 4). The retained FH staining in internal endothelial cells and the diffuse 2SC positivity provided unequivocal IHC evidence. The wild-type p53 pattern and low Ki-67 index helped exclude leiomyosarcoma and other high-grade mimics.

From a management perspective, this case necessitates a paradigm shift. The successful surgical treatment of the fibroids transitions into a lifelong scenario of cancer surveillance and genetic counseling. The absence of a positive family history, as seen here, is not reassuring, as *de novo* FH mutations are well-documented, and penetrance can be variable (8). The recommended annual renal surveillance with MRI is non-negotiable, given the potential for HLRCC-associated renal cell carcinoma to be aggressive and metastasize even at a small size (9). Our decision to initiate surveillance based solely on pathology is endorsed by consensus guidelines, which state that the finding of an FH-deficient uterine leiomyoma, irrespective of germline mutation status, warrants HLRCC-directed renal screening (5).

The differential diagnosis for uterine smooth muscle tumors with atypia includes atypical leiomyoma, leiomyosarcoma, and smooth muscle tumors of uncertain malignant potential. The IHC profile (FH-/2SC+) is pathognomonic for FH-deficiency and effectively distinguishes it from these entities. Furthermore, this case adds to the limited literature on HLRCC in non-Caucasian populations (10), reinforcing that this syndrome exists across ethnicities and should be considered universally in the appropriate clinical context.

We acknowledge several limitations of this report. The absence of germline FH testing prevents definitive distinction between a hereditary and a somatic etiology of the FH-deficient leiomyoma. The short-term follow-up limits our ability to comment on long-term renal outcomes. Additionally, while the photomicrographs illustrate the salient features, some of the classic nuclear details of FH-deficient leiomyoma are subtle in this particular case. Nevertheless, the IHC diagnosis is unequivocal and drives the clinical action. Long-term surveillance data, including renal MRI findings and cascade testing, will be collected and reported in future publications.

## 4 Conclusion

The diagnosis of an FH-deficient leiomyoma is a pivotal event in the management of young women with multiple uterine fibroids, particularly those with a short history of mass and rapidly progressive symptoms. Regardless of whether the underlying genetic defect is germline or somatic, the pathological finding mandates a paradigm shift from routine gynecologic care to a coordinated, multidisciplinary strategy encompassing genetics, urology-oncology, and dermatology. Gynecologists must be aware of this entity, and pathologists play a critical role in triggering the appropriate diagnostic workup through judicious use of FH/2SC immunohistochemistry. This case serves as a compelling reminder that behind a common gynecologic presentation may lie a rare but potentially life-saving diagnosis of a potential hereditary cancer syndrome, and that even in settings with limited access to molecular testing, evidence-based surveillance can and should be implemented.

## Data Availability

The original contributions presented in the study are included in the article/**Supplementary Material**, further inquiries can be directed to the corresponding author/s.

## Supplementary material

Supplementary Figure 1 Clinical timeline summarizing the key events from incidental pelvic mass detection to postoperative follow-up and surveillance planning.
